# Resuscitation of Frozen Animals

**Published:** 1882-03

**Authors:** 


### RESUSCITATION OF FROZEN ANIMALS.

A conflict of opinion exists between experimenters on the one hand and clinical surgeons on the other as to the best method of resuscitating frozen animals (including human beings). While the latter almost without exception advocate the gradual introduction of heat, the former (Beck, Horwat and Jacoby) claim that it should be applied rapidly. In order to decide this question, Laptschinski has performed careful experiments upon dogs in the clinic of Prof. Manassein. The results were confirmatory of his views on the subject, and are summarized as follows: Of twenty animals treated by the method of gradual resuscitation in a cold room, fourteen died; of twenty introduced at once into a warm apartment, eight perished, while of twenty placed immediately in a hot bath all recovered.—*Internat. Surg. Record.*

